# Molecular Mimicry and HLA Polymorphisms May Drive Autoimmunity in Recipients of the BNT-162b2 mRNA Vaccine: A Computational Analysis

**DOI:** 10.3390/microorganisms11071686

**Published:** 2023-06-28

**Authors:** Rossella Talotta

**Affiliations:** Rheumatology Unit, Department of Clinical and Experimental Medicine, University Hospital “G. Martino”, 98124 Messina, Italy; rtalotta@unime.it; Tel.: +39-0902212028

**Keywords:** adaptive immunity, autoimmunity, BNT-162b2 vaccine, COVID-19, HLA, SARS-CoV-2, spike

## Abstract

Background: After the start of the worldwide COVID-19 vaccination campaign, there were increased reports of autoimmune diseases occurring de novo after vaccination. This in silico analysis aimed to investigate the presence of protein epitopes encoded by the BNT-162b2 mRNA vaccine, one of the most widely administered COVID-19 vaccines, which could induce autoimmunity in predisposed individuals. Methods: The FASTA sequence of the protein encoded by the BNT-162b2 vaccine served as the key input to the Immune Epitope Database and Analysis Resource. Linear peptides with 90% BLAST homology were selected, and T-cell, B-cell, and MHC-ligand assays without MHC restriction were searched and analyzed. HLA disease associations were screened on the HLA-SPREAD platform by selecting only positive markers. Results: By 7 May 2023, a total of 5693 epitopes corresponding to 21 viral but also human proteins were found. The latter included CHL1, ENTPD1, MEAF6, SLC35G2, and ZFHX2. Importantly, some autoepitopes may be presented by HLA alleles positively associated with various immunological diseases. Conclusions: The protein product of the BNT-162b2 mRNA vaccine contains immunogenic epitopes that may trigger autoimmune phenomena in predisposed individuals through a molecular mimicry mechanism. Genotyping for HLA alleles may help identify individuals at risk. However, further wet-lab studies are needed to confirm this hypothesis.

## 1. Introduction

With more than 760,000,000 confirmed cases and 6,000,000 deaths worldwide [1], the CoronaVirus Disease-19 (COVID-19) pandemic appeared as one of the most impressive epidemics of this century and led to unprecedented efforts to pharmacologically counteract the further spread of infection and the most severe consequences of the disease. Sequencing of the Severe Acute Respiratory Syndrome CoronaVirus (SARS-CoV)-2 genome enabled the development of mRNA, viral vector, and inactivated vaccines and, more recently, peptide vaccines against the original and newer viral variants. They all aim to generate an immune response against the SARS-CoV-2 spike protein, an envelope glycoprotein that allows the virus to enter target cells by binding to the receptor angiotensin-converting enzyme 2 (ACE 2) [2]. These vaccines were quickly made available and distributed to people around the world through massive vaccination campaigns. The World Health Organization (WHO) estimates that a total of 13,350,487,934 doses of vaccine have been administered as of 10 May 2023 [1]. Commercially available anti-SARS-CoV-2 vaccines have an acceptable overall safety profile. However, since their introduction in December 2020, a cumulative number of reports have described cases of association with autoimmune phenomena in previously otherwise healthy individuals. These events include connective tissue diseases, autoimmune arthritis, myocarditis, and vasculitis [3,4]. On the other hand, anti-SARS-CoV-2 vaccines have been shown to be safe in patients with previously diagnosed autoimmune diseases in both clinical trials and real-life studies [5,6,7,8,9,10]. These seemingly contradictory data may be explained by a different pharmacological background of the two cohorts studied (no immunosuppressive therapy in patients with de novo developing diseases). Indeed, studies have shown that concomitant use of steroids and immunosuppressants or biologic agents such as methotrexate (MTX), mycophenolate mofetil (MMF), and rituximab (RTX) can impair the humoral response to anti-SARS-CoV-2 vaccines and, thus, the risk of immune-mediated adverse events [10,11,12]. On the other hand, it is well-established that vaccines can induce aberrant immune responses in otherwise healthy, genetically predisposed individuals, leading in some cases to the development of full-blown autoimmune diseases [13]. Underlying mechanisms include molecular mimicry, activation of pattern recognition receptors (PRRs), polyclonal stimulation of B cells, autoinflammation, and type I interferon (IFN-I) production. The major histocompatibility complex (MHC), which in humans is encoded by genes in the polymorphic human leukocyte antigen (HLA) loci, has been shown to contribute up to 50% of the genetic risk for autoimmune diseases [14,15]. HLA proteins are involved in the presentation of epitopes to T lymphocytes, and thus play a crucial role in modulating the immune response against foreign and self-proteins. Linkage and association studies have established a close relationship between certain HLA alleles and type 1 diabetes, ankylosing spondylitis, rheumatoid arthritis, celiac disease, as well as some infections and cancer [14]. Overall, polymorphic variants of the class I HLA alleles may promote an atypical cytotoxic T-lymphocyte response, whereas those found in the class II HLA may favor humoral or granulomatous diseases. Because of the high variability of HLA alleles across ethnic groups, the association of specific alleles and haplotypes with human disease is often inconsistent, meaning that HLA alleles may be a risk factor for disease susceptibility in one population and protective for the same disease in other populations [14].

It is possible that autoimmunity following COVID-19 vaccine administration may be due to a genetically favorable background represented primarily by specific HLA alleles or haplotypes. In this scenario, autoimmune phenomena could be interpreted as the result of the interaction between the genetic susceptibility of the vaccinated individual and the vaccine itself acting as an environmental trigger. The molecular similarity between vaccine-encoded epitopes and human epitopes could lead to cross-reactivity of immune cells or antibodies to foreign and self-antigens [16]. Although several studies show that polymorphisms of HLA loci may influence COVID-19 morbidity and mortality or affect the immune response to vaccines [17], it is so far unclear whether they could also lead to the breakdown of immune tolerance to self-epitopes with molecular mimicry to anti-SARS-CoV-2 vaccines.

Therefore, the aim of this work was to investigate in silico the presence of protein epitopes encoded by the BNT-162b2 mRNA vaccine, one of the most commonly administered COVID-19 vaccines, which may possibly induce autoimmunity in genetically predisposed individuals.

## 2. Materials and Methods

### 2.1. IEDB Analysis

The FASTA sequence of the spike protein encoded by the BNT-162b2 mRNA vaccine is publicly available at http://genome.ucsc.edu accessed on 7 May 2023 (http://genome-euro.ucsc.edu/cgi-bin/hgTrackUi?db=wuhCor1&c=NC_045512v2&g=vaccines, accessed on 7 May 2023). It was used as a key input to the Immune Epitope Database and Analysis Resource (IEDB) (www.iedb.org, accessed on 7 May 2023) [18]. The IEDB is an open access platform that contains the results of more than 1.6 million experiments on adaptive immune response epitopes drawn from the current literature. The database is regularly updated every two weeks by adding newer results from PubMed content. It has an intuitive user interface and additional tools. Peptidic epitopes belonging to different species, including viruses, are obtained by combining UniProt’s proteomic data and are also regularly implemented [19]. For this analysis, the official IEDB database was consulted, selecting the following options: linear peptides with 100% and 90% BLAST homology to the BNT-162b2 vaccine FASTA protein sequence; epitope source: none; host: Homo sapiens (human). The search was limited to all positive tests without MHC restriction and without disease association. At the time of the search, the database had been updated on 7 May 2023.

### 2.2. TepiTool Analysis

To test the MHC-binding affinity of detected self-epitopes, the TepiTool from the IEDB resources was consulted [20]. The TepiTool allows prediction of peptides binding to class I and class II MHC molecules and provides an estimate of binding affinity using the consensus percentile rank. For this analysis, the FASTA protein sequence of the epitopes of interest was used to perform a dual search for class I and class II MHC alleles in human species. Nine-mer peptides were analyzed for their binding affinity to class I HLA alleles selected from a list of all available alleles. The recommended default settings for prediction and selection of optimal peptides were maintained (low number of peptides with deletion of duplicates, predicted consensus percentile rank < 1, prediction method: IEDB recommended, conservancy analysis: no). Alleles of the class II HLA, predicted to bind to 15-mer peptides, were instead selected from a panel of the 26 most frequent alleles (DRB1*01:01, DRB1*03:01, DRB1*04:01, DRB1*04:05, DRB1*07:01, DRB1*08:02, DRB1*09:01, DRB1*11:01, DRB1*12:01, DRB1*13:02, DRB1*15:01, DRB3*01:01, DRB3*02:02, DRB4*01:01, DRB5*01:01, DPA1*01/DPB1*04:01, DPA1*01:03/DPB1*02:01, DPA1*02:01/DPB1*01:01, DPA1*02:01/DPB1*05:01, DPA1*03:01/DPB1*04:02, DQA1*01:01/DQB1*05:01, DQA1*01:02/DQB1*06:02, DQA1*03:01/DQB1*03:02, DQA1*04:01/DQB1*04:02, DQA1*05:01/DQB1*02:01, and DQA1*05:01/DQB1*03:01) and binding affinity was calculated using the IEDB recommended prediction method (predicted consensus percentile rank < 10, low number of peptides, removal of duplicate peptides, and no conservancy analysis).

### 2.3. HLA-SPREAD Analysis

The associations between HLA alleles predicted to bind to epitopes of the protein encoded by the BNT-162b2 vaccine and human disease were screened on the HLA-SPREAD platform (https://hla-spread.igib.res.in, accessed on 7 May 2023) [21]. The HLA-SPREAD platform consists of a huge database that collects evidence from more than 28 million peer-reviewed articles published on MEDLINE. It summarizes the results of studies on associations between class I and II HLA allele variants and diseases or adverse drug reactions and responses in an intuitive way and provides other useful information, including geographic locations of the cohorts studied and HLA single-nucleotide polymorphisms (SNPs). For this analysis, only positive classes of associations with immune-mediated diseases were considered, indicating an increased risk for the disease in carriers of the allele of interest.

## 3. Results

### 3.1. IEDB Analysis Results

While the search for exact matches yielded no results, the search for epitopes showing 90% BLAST homology to the BNT-162b2 mRNA vaccine-encoded protein sequence yielded a total of 5693 records, as shown in Table S1. Results were, overall, obtained from 4170 T-cell assays, 8965 B-cell assays, and 4206 MHC-ligand assays. Specifically, 4432 epitopes belonged to SARS-CoV-2 spike glycoprotein, whereas the remaining were found in other antigens, including other non-coronavirus and human proteins, as shown in Table 1. Complementary receptors consisted of 17,996 T-cell receptors (TCRs) and 19 B-cell receptors (BCRs).

**Figure 1:**
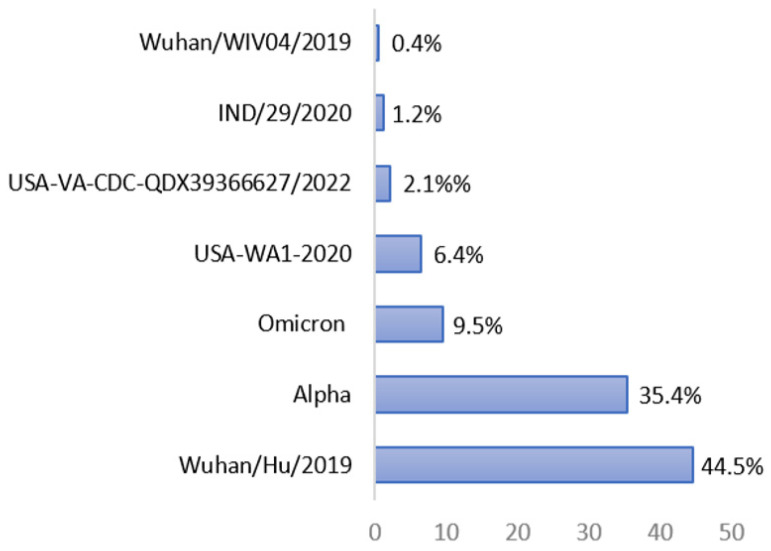
Percentage of BNT-162b2 vaccine-90% BLAST-homolog epitopes found in the spike protein of different SARS-CoV-2 strains according to IEDB analysis (last update on 7 May 2023).

For the SARS-CoV-2 strain, most epitopes (44.5%) were derived from the spike protein of the original SARS-CoV-2 Wuhan/Hu/2019 virus, followed by the SARS-CoV-2 Alpha strain (35.4%), the SARS-CoV-2 Omicron strain (9.5%), the SARS-CoV-2 USA-WA1-2020 strain (6.4%), the SARS-CoV-2 USA-VA-CDC-QDX39366627/2022 strain (2.1%), the SARS-CoV-2 IND/29/2020 strain (1.2%), and the Wuhan/WIV04/2019 strain (0.4%), as shown in Figure 1.

Interestingly, the BNT-162b2 mRNA vaccine appeared to contain epitopes showing 90% BLAST homology with human proteins, namely ectonucleoside triphosphate diphosphohydrolase 1 (ENTPD1), neuronal cell adhesion molecule L1-like protein (CHL1), solute carrier family 35 member G2 (SLC35G2), chromatin modification-related protein MYST /Esa1-associated factor 6 (MEAF6), and zinc finger homeobox protein 2 (ZFHX2). Specific HLA-binding alleles were available for some of them, as shown in Table 2.

### 3.2. HLA-SPREAD Analysis Results

When available, HLA alleles or haplotypes that bind to self-epitopes were searched for positive associations with human autoimmune diseases in the HLA-SPREAD database. The results are shown in Table 3. Briefly, some epitopes belonging to MEAF6, CHL1, and ZHX2 were reported to be potentially presented by either class I or class II HLA alleles for which a clear association with autoimmune diseases has been described. These include multiple sclerosis, type 1 diabetes, psoriasis, rheumatoid arthritis, seronegative arthritis, systemic lupus erythematosus, and systemic sclerosis.

### 3.3. TepiTool Analysis Results

Indeed, the affinity of the MHC cleft for the peptide may influence the capacity to stimulate the immune system. By using the TepiTool program, an analysis was conducted to predict the binding affinity of peptides from CHL1, MEAF6, and ZFHX2 to MHC molecules for which specific binding was demonstrated. As shown in Table 4, peptides within the MEAF6 and CHL1 proteins showed the highest binding affinity to the class I HLA allele HLA-A*24:02, whereas the epitope PPEAEVQALILLDEE belonging to ZHX2 had the largest predicted interaction with the class II HLA allele HLA-DRB1*11:01.

## 4. Discussion

Notwithstanding the limitations arising from the bioinformatic nature of the analysis, this work provides insights into the possible pathogenic mechanism linking one of the most widely used COVID-19 vaccines to autoimmune disease risk, which may pave the way for further research in this area.

The BNT-162b2 mRNA vaccine encodes the full-length SARS-CoV-2 spike protein, derived from the *S* gene sequence of the SARS-CoV-2 isolate Wuhan-Hu-1 (GenBank MN908947.3) [22], which was stabilized in the prefusion conformation via substitution of the amino acid residues 986 and 987 with proline.

According to the results of this study, the protein product of the BNT-162b2 mRNA vaccine may contain epitopes capable of mimicking self-epitopes or binding to HLA alleles, which in turn have been associated with the risk of certain autoimmune diseases in certain ethnic groups.

Such epitopes can be recognized by TCRs and trigger the activation of effector T cells. It is known that polymorphic HLA genes can encode isoforms of MHC molecules that have amino acid substitutions at crucial positions such as peptide binding sites [23]. These variants may centrally influence thymic selection or affect the binding affinity of epitopes peripherally, ultimately increasing the repertoire of recognized allo- and autoepitopes and expanding the pool of reactive T and B cells. Consequently, polymorphisms within HLA loci may enhance immune defenses against pathogens but also increase the risk of developing autoimmune diseases [21]. HLA genes may actually affect the survival of T cells with specific patterns in the complementarity determining region 3 (CDR3) of the TCR, characterized by a higher ability to present autoepitopes [23].

The IEDB database search resulted in a total of 5693 epitopes tested in 4130 T-cell assays, 8965 B-cell assays, and 4206 MHC-ligand assays. In addition, 17,996 complementary TCRs and 19 BCRs were recorded. It is important to note that these results represent the current state of research, which was updated in May 2023. It is expected that the above numbers will increase in the coming months as more information becomes available from newer experiments.

According to this analysis, the epitopes within the spike protein encoded by the BNT-162b2 mRNA vaccine belong either to SARS-CoV-2 or to other coronaviruses. These data are not surprising given the overlap in molecular structure of the spike protein between SARS-CoV-2 and other coronaviruses and the 76% identity and 86% similarity between SARS-CoV-2 and SARS-CoV spike [24,25]. Importantly, the BNT-162b2 vaccine appears to encode a protein that has epitopes with 90% BLAST homology to other non-coronaviruses as well as human proteins. Therefore, it should be better clarified what role previous cross-reactive infections play in priming T cells, which may potentially affect the immunogenicity of COVID-19 vaccines [25].

Regarding self-antigens, most of the BNT-162b2 spike-overlapping human epitopes are located in ENTPD1 (or CD39), an enzyme that can be detected in monocytes, dendritic cells (DCs), neutrophils, B lymphocytes, and some natural killer (NK) and T cell subsets [26]. ENTPD1 catalyzes the phosphohydrolysis of extracellular adenosine triphosphate (eATP) and diphosphate (eADP) to adenosine monophosphate (AMP). eATP is usually released under inflammatory stress conditions and cell injury, while AMP has immunomodulatory properties. Interestingly, increased production of eATP can lead to autoimmune diseases such as type 1 diabetes or neuroinflammation, migraine, and neuropathic pain [27,28]. Strikingly, several cases of de novo type 1 diabetes have been reported in the Japanese population after COVID-19 mRNA vaccination [29,30,31]. IEDB analysis also revealed 90% homology with the human CHL1. This result is of particular interest because the protein is involved in synaptic plasticity and central and peripheral nociception [32]. Proteomic studies of cerebrospinal fluid (CSF) revealed dysregulated levels of this protein in patients with fatigue and multiple sclerosis or neuropathic pain [32,33]. Given the risk of neurological side effects of COVID-19 vaccines [34], a molecular mimicry mechanism between spike and CHL1, at least for the BNT-162b2 formulation, could be hypothesized. Moreover, epitopes in the BNT-162b2 vaccine may match epitopes of ZFHX2, a DNA-binding protein whose gene mutation has been associated with pain insensitivity in small diameter sensory neurons [35]. On the other hand, the role of MEAF6, a component of NuA4 histone acetyltransferase [36], and SLC35G2, a Golgi transporter [37], as potential autoantigens is unknown. Recently, a preclinical experiment in mouse models demonstrated that disruption of the NPAS4-NuA4 complex in activated neurons can impair stimulus-induced gene repair and affect the expression of genes that mediate somatic inhibition recruitment, which in turn leads to excessive neuronal excitation that can result in genome instability [38]. These events can lead to cognitive and sensory processing impairments and accelerated aging. Indeed, mutations of genes encoding for subunits of the NPAS4-NuA4 complex have been described in neurodevelopmental and autism spectrum disorders. Therefore, although not yet supported by experimental data, it could be hypothesized that BNT-162b2 vaccine-mediated cellular or humoral cross-reactivity toward the MEAF6 subunit could reduce the neuroprotective activity of the NPAS4-NuA4 complex and promote the risk of defective neuronal development in very young subjects and neurodegenerative disorders in adults.

**Figure 2:**
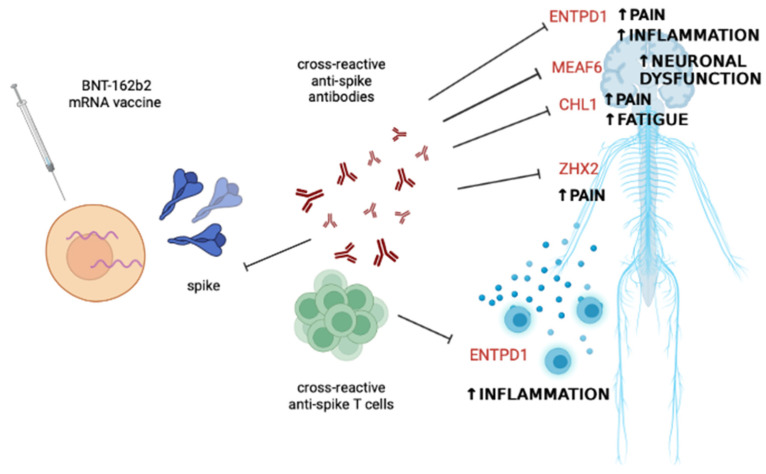
Potential scenario explaining some of the immune-mediated side effects following BNT-162b2 mRNA vaccine administration. Cross-reactivity between the epitopes present in the encoded spike protein and self-epitopes of proteins mostly involved in nervous system function and control of inflammation may account for cases of fatigue, neuroinflammation, neuronal dysfunction, central and peripheral pain, and systemic inflammation. Abbreviations: CHL1, neural cell adhesion molecule L1-like protein; ENTPD1, ectonucleoside triphosphate diphosphohydrolase 1; MEAF6, MYST/Esa1-associated factor 6; ZFHX2, zinc finger homeobox 2. The figure was created with BioRender.com, accessed on 18 June 2023.

Based on these data, it can be assumed that the autoimmune phenomena reported in the literature after vaccination may be due to cross-reactive antibodies or T cells against self-epitopes that have homologies with epitopes of the BNT-162b2 vaccine spike, as shown in Figure 2.

In addition to molecular homology and cross-reactivity, allelic variants of the HLA are of paramount importance in the development of autoimmunity. The results of this computational analysis show that some epitopes of the protein product of the BNT-162b2 mRNA vaccine could be efficiently presented by nucleated cells and antigen-presenting cells (APCs) through preferential binding of some class I or class II HLA molecules. Importantly, research shows that HLA polymorphisms may also dictate the clinical course of COVID-19 or response to vaccines by influencing epitope presentation to protective T cells [39,40,41,42,43,44], as shown in Table 5.

Some of the HLA alleles that showed associations with COVID-19 progression or response to the vaccine were predicted in this analysis to bind to self-epitopes showing 90% BLAST homology with the BNT-162b2 mRNA vaccine protein. For example, the HLA-DRB1*15:01 allele, predicted to bind epitopes of MEAF and ZFHX2, was positively correlated with enhancement of both anti-spike B and T cell responses in Caucasian and Japanese BNT-162b2 mRNA vaccine recipients [39,41,42]. Conversely, the allele HLA-A*24:02, which possibly recognizes epitopes of CHL1, was negatively related to the production of anti-spike antibodies after BNT-162b2 mRNA vaccination in an Italian study [41]. However, it is unclear whether T-cell responses are equally impaired in carriers of this HLA allele. Interestingly, the data from this computational analysis suggest that both the HLA-DRB1*15:01 and HLA-A*24:02 alleles may have a very high binding affinity for autoepitopes. In other words, carriers of the HLA-DRB1*15:01 and HLA-A*24:02 alleles may develop a cross-reactive immune response after BNT-162b2 vaccination that may neutralize the function of ZFHX2, MEAF6, and CHL1 or lead to direct tissue damage, mainly manifested by fatigue, pain, and other neurological symptoms.

Notwithstanding these intriguing results, most data from either clinical trials or real-life cohort studies of people with autoimmune diseases do not evidence an increased safety risk after COVID-19 vaccine administration in terms of disease flares or worsening, as shown in Table 6 [5,6,7,8,9,10,11,12,45,46]. However, these results may be confounded by concomitant immunosuppressive treatments that limit the magnitude of the immune response to both the vaccine and self-epitopes [47]. Other reasons for such a discrepancy could be that safety was usually not a primary endpoint of the studies; adverse events were reported via questionnaires or over a very short period of time (from a few days to a few weeks), whereas autoimmune reactions could take longer, and in most cases were mentioned as general manifestations not necessarily related to an autoimmune mechanism. In addition, the sample of patients analyzed was often too small or too different from the control group, and a control group of unvaccinated patients was often missing. Indeed, disease relapse or worsening has been reported in 3% to 19% of patients with autoimmune diseases, including multiple sclerosis and systemic lupus erythematosus [4]. In addition, neurological complications following COVID-19 vaccination have been observed primarily in individuals with a history of autoimmunity [4].

## 5. Conclusions

In summary, this computational study shows that the anti-SARS-CoV-2 BNT-162b2 vaccine encodes a spike protein that has multiple epitopes that can cross-react with human proteins or bind to HLA alleles or haplotypes that are significantly associated with autoimmune diseases in different geographic areas.

The major limitation of this analysis is its bioinformatic nature. The results were extrapolated from preclinical experiments with linear peptides that are regularly updated on the IEDB platform. Thus, they reflect the current scientific knowledge and it is expected that the panorama will be enriched with new data in the coming months due to the enormous ongoing research in this field. Moreover, this computational model could not account for the post-translational modifications of the spike protein that may actually affect immunogenicity. Indeed, it is known that the SARS-CoV-2 spike protein is highly N-glycosylated, which may mask epitope sites [48]. The virus could also prevent translation of host proteins and affect HLA presentation and ubiquitin-mediated proteasome degradation [17].

Another limitation of this study is the lack of an assessment of the contribution of HLA-linked genes [14] or non-HLA genes to autoimmunity risk.

In addition, the results of the analysis suggest that autoimmune phenomena may occur after COVID-19 vaccination, but also after SARS-CoV-2 and other viral infections, which may add up to an immunogenic effect of the vaccine as a “second shot”.

Finally, this analysis focused on the FASTA protein sequence of the vaccine BNT-162b2, which encodes the wild-type spike protein of SARS-CoV-2. Due to the emergence of SARS-CoV-2 variants and mutations in the viral spike gene, research is now shifting to the formulation of next-generation vaccines that can provide broader protection against both the Wuhan strain of SARS-CoV-2 and other variants of concern (VOCs) [49]. The presence of epitopes in these new formulations able to interact with specific HLA alleles and favor the onset of autoimmune phenomena should be investigated. In particular, research has shown that some HLA alleles may have different binding affinities to the original Wuhan, Delta, and Omicron strains [50,51]. Similarly, it remains to be investigated whether these results can apply to other widely used vaccine formulations. Indeed, studies have shown that epitopes from the protein product of the anti-SARS-CoV-2 mRNA-1273 vaccine are presented in an MHC-restricted manner, potentially eliciting an adverse immune response in carriers of risk alleles [52].

It is expected that further preclinical and clinical research will address some of these caveats in the future and help to better define the immunopathogenic role of COVID-19 vaccines in the context of autoimmune diseases.

## Figures and Tables

**Table 1:** List of the antigens containing epitopes also found in the spike protein encoded by the BNT-162b2 mRNA vaccine according to IEDB analysis (last update on 7 May 2023). Abbreviations: HCV, hepatitis C virus; IRI, Internationalized Resource Identifier; MERS, Middle East respiratory syndrome; SARS-CoV, severe acute respiratory syndrome coronavirus.

Antigen Name	Antigen IRI	Organism Name	Organism IRI	Epitopes (n°)	Assays (n°)	References (n°)
Envelope glycoprotein gp62	http://www.uniprot.org/uniprot/P03381, accessed on 7 May 2023	Primate T-lymphotropic virus 1	http://purl.obolibrary.org/obo/NCBITaxon_1944, accessed on 7 May 2023	7	23	12
Spike glycoprotein	http://www.uniprot.org/uniprot/P59594, accessed on 7 May 2023	SARS-CoV1	https://ontology.iedb.org/taxon/10002316, accessed on 7 May 2023	913	1624	62
Genome polyprotein	http://www.uniprot.org/uniprot/P27958, accessed on 7 May 2023	HCV	http://purl.obolibrary.org/obo/NCBITaxon_1110, accessed on 7 May 2023	1	4	1
Solute carrier family 35 member G2	http://www.uniprot.org/uniprot/Q8TBE7, accessed on 7 May 2023	Homo sapiens	http://purl.obolibrary.org/obo/NCBITaxon_9606, accessed on 7 May 2023	1	1	1
Ectonucleoside triphosphate diphosphohydrolase 1 (UniProt:P49961)	http://www.uniprot.org/uniprot/P49961, accessed on 7 May 2023	Homo sapiens	http://purl.obolibrary.org/obo/NCBITaxon_9606, accessed on 7 May 2023	4	11	9
Neural cell adhesion molecule L1-like protein (UniProt:O00533)	http://www.uniprot.org/uniprot/O00533, accessed on 7 May 2023	Homo sapiens	http://purl.obolibrary.org/obo/NCBITaxon_9606, accessed on 7 May 2023	2	3	3
Chromatin modification-related protein MEAF6 (UniProt:Q9HAF1)	http://www.uniprot.org/uniprot/Q9HAF1, accessed on 7 May 2023	Homo sapiens	http://purl.obolibrary.org/obo/NCBITaxon_9606, accessed on 7 May 2023	2	2	2
Spike glycoprotein	http://www.uniprot.org/uniprot/Q3LZX1, accessed on 7 May 2023	Other SARS	https://ontology.iedb.org/taxon/10002383, accessed on 7 May 2023	116	163	2
Other SARS-CoV1 protein	https://ontology.iedb.org/taxon-protein/10002316-other, accessed on 7 May 2023	SARS-CoV1	https://ontology.iedb.org/taxon/10002316, accessed on 7 May 2023	18	35	1
Other SARS protein	https://ontology.iedb.org/taxon-protein/10002383-other, accessed on 7 May 2023	Other SARS	https://ontology.iedb.org/taxon/10002383, accessed on 7 May 2023	3	6	1
Spike glycoprotein	http://www.uniprot.org/uniprot/A0A140H1H1, accessed on 7 May 2023	Human coronavirus HKU1	http://purl.obolibrary.org/obo/NCBITaxon_2900, accessed on 7 May 2023	3	3	2
Replicase polyprotein 1ab	http://www.uniprot.org/uniprot/K9N7C7, accessed on 7 May 2023	MERS-related coronavirus	http://purl.obolibrary.org/obo/NCBITaxon_1335, accessed on 7 May 2023	4	4	1
Spike glycoprotein	http://www.uniprot.org/uniprot/K9N5Q8, accessed on 7 May 2023	MERS-related coronavirus	http://purl.obolibrary.org/obo/NCBITaxon_1335, accessed on 7 May 2023	32	59	2
Spike glycoprotein	http://www.uniprot.org/uniprot/P0DTC2, accessed on 7 May 2023	SARS-CoV2	http://purl.obolibrary.org/obo/NCBITaxon_2697049, accessed on 7 May 2023	4432	14837	225
Replicase polyprotein 1ab	http://www.uniprot.org/uniprot/P0C6X7, accessed on 7 May 2023	SARS-CoV1	https://ontology.iedb.org/taxon/10002316, accessed on 7 May 2023	9	9	1
Spike glycoprotein	http://www.uniprot.org/uniprot/A0A0P0G321, accessed on 7 May 2023	Human coronavirus NL63	http://purl.obolibrary.org/obo/NCBITaxon_2779, accessed on 7 May 2023	1	5	2
Spike glycoprotein	http://www.uniprot.org/uniprot/A0A0P0K6L9, accessed on 7 May 2023	Human coronavirus 229E	http://purl.obolibrary.org/obo/NCBITaxon_11137, accessed on 7 May 2023	1	8	2
Spike protein	http://www.uniprot.org/uniprot/F1DB20, accessed on 7 May 2023	Chaerephon bat coronavirus/Kenya/KY22/2006	http://purl.obolibrary.org/obo/NCBITaxon_9839, accessed on 7 May 2023	1	4	1
Zinc finger homeobox protein 2	http://www.uniprot.org/uniprot/Q9C0A1, accessed on 7 May 2023	Homo sapiens	http://purl.obolibrary.org/obo/NCBITaxon_9606, accessed on 7 May 2023	1	1	1
Spike glycoprotein	http://www.uniprot.org/uniprot/A0A059VFK8, accessed on 7 May 2023	Porcine epidemic diarrhea virus	http://purl.obolibrary.org/obo/NCBITaxon_2829, accessed on 7 May 2023	3	6	1
Spike glycoprotein	http://www.uniprot.org/uniprot/A0A060A825, accessed on 7 May 2023	Coronavirus HKU15	http://purl.obolibrary.org/obo/NCBITaxon_1965089, accessed on 7 May 2023	2	7	2

**Table 2:** Human protein epitopes sharing 90% BLAST homology with the spike protein encoded by the BNT-162b2 mRNA vaccine, according to IEDB analysis (last update on 7 May 2023). Abbreviations: CHL1, neuronal cell adhesion molecule L1-like protein; DDA, data-dependent acquisition; ENTPD1, ectonucleoside triphosphate diphosphohydrolase 1; ERAP2, endoplasmic reticulum aminopeptidase 2; HLA, human leukocyte antigen; LC/MS, liquid chromatography/mass spectrometry MEAF6, MYST /Esa1-associated factor 6; MHC, major histocompatibility complex; SLC35G2, solute carrier family 35 member G2; UV, ultraviolet; ZFHX2, zinc finger homeobox protein 2.

Epitope	Source Molecule	MHC Type Presentation	Antigen Processing	Assay
FEMTLPFQQF	ENTPD1		HLA class I-bound peptides were isolated from human C1R B cell lines (stably expressing B27, B39, or B40) using immunoaffinity chromatography and analyzed with DDA on an Orbitrap-XL mass spectrometer	cellular MHC/mass spectrometry
MYGNIIRGW	chromosome 1 open reading frame 149, isoform CRA_c			secreted MHC/mass spectrometry
LPFQQFEI	ENTPD1 isoform 2		HLA-B*51:01 expressing 721.221 cells were used as the source for the HLA molecule	cellular MHC/mass spectrometry
TLPFQQFEI	ENTPD1 isoform 7			cellular MHC/mass spectrometry
LPFQQFEI	ENTPD1 isoform 7			cellular MHC/mass spectrometry
LPFQQFEI	ENTPD1 isoform 1		CIR B cells were transfected with a single HLA-A (A*01:01, A*02:03, A*02:04, A*02:07, A*03:01, A*24:02, A*31:01, or A*68:02) or HLA-B allele (B*07:02, B*08:01, B*15:02, B*27:05, B*44:02, B*51:01, B*57:01, B*57:03, or B*58:01). HLA–peptide complexes were immunoaffinity purified from cell lysates using anti-HLA class I mAb W6/32. Bound complexes were acid-eluted and the peptide ligands were isolated and analyzed using LC-MS/MS	cellular MHC/mass spectrometry
FEMTLPFQQF	ENTPD1 isoform 1		Stable C1R transfectants expressing HLA-B*40:02 were used for immunopeptidomics studies. The cells were either wild-type or ERAP2 knockouts	cellular MHC/mass spectrometry
LPFQQFEI	ENTPD1 isoform 1			cellular MHC/mass spectrometry
TLPFQQFEI	ENTPD1		The HLA class I-deficient B721.221 cell line was transfected with the HLA allele expression vectors for single-HLA alleles	mass spectrometry
LPFQQFEI	ENTPD1		The HLA class I-deficient B721.221 cell line was transfected with the HLA allele expression vectors for single-HLA alleles	mass spectrometry
TKRFEMTLPFQQFE	ENTPD1			cellular MHC/mass spectrometry
KPQSAVYSTGSNGIL	CHL1			cellular MHC/mass spectrometry
TAAFLGVYYALDK	SLC35G2			cellular MHC/mass spectrometry
LEDTQMYGNIIRGWDRYLTNQKNSN	chromatin modification-related protein MEAF6	HLA-A*24:02; HLA-A*25:01; HLA-B*18:01; HLA-B*41:01; HLA-C*12:03; HLA-C*17:01; HLA-DPA1*01:03/DPB1*02:01; HLA-DPA1*01:03/DPB1*09:01; HLA-DPA1*02:01/DPB1*02:01; HLA-DPA1*02:01/DPB1*09:01; HLA-DQA1*01:02/DQB1*02:02; HLA-DQA1*01:02/DQB1*06:02; HLA-DQA1*03:03/DQB1*02:02; HLA-DQA1*03:03/DQB1*06:02; HLA-DRB1*04:05; HLA-DRB1*15:01; HLA-DRB4*01:03; HLA-DRB5*01:01		cellular MHC/mass spectrometry
KPQSAVYSTGSNGILL	CHL1	HLA-A*24:02; HLA-A*30:01; HLA-B*13:02; HLA-B*35:08; HLA-C*04:01; HLA-C*06:02; HLA-DPA1*01:03/DPB1*03:01; HLA-DPA1*01:03/DPB1*04:02; HLA-DQA1*02:01/DQB1*02:02; HLA-DRB1*07:01; HLA-DRB4*01:03		cellular MHC/mass spectrometry
FEMTLPFQQF	ENTPD1		C1R cells modified to express HLA-B*15:02 were treated with carbamazepine	cellular MHC/mass spectrometry
PPEAEVQALILLDEE	ZFHX2	HLA-DRB1*11:01; HLA-DRB1*15:01; HLA-DQB1*03:01; HLA-DQB1*06:02; HLA-DPB1*01:01; HLA-DPB1*04:01	Resected human lung parenchymal tissues from three donors were either mock-infected with UV-inactivated influenza or infected with A/H3N2/Wisconsin/67/2005 or A/X-31 H3N2, and then subjected to ligand elution	cellular MHC/mass spectrometry
KPQSAVYSTGSNGIL	CHL1	HLA-DRB1*07:01; HLA-DRB4*01:03		cellular MHC/mass spectrometry

**Table 3:** MHC-restricted epitopes of BNT-162b2 vaccine and potentially associated immunological conditions in different geographic groups. Abbreviations: anti-gbm, anti-glomerular basement membrane; CHL1, neural cell adhesion molecule L1-like protein; HLA, human leukocyte antigen; MEAF6, MYST/Esa1-associated factor 6; NA, not available; ZFHX2, zinc finger homeobox protein 2.

Epitope	Human Protein	HLA-Presenting Molecule	Associated Disease	Ethnic Group
LEDTQMYGNIIRGWDRYLTNQKNSN	MEAF6	HLA-A*24:02	arthritis	Basque
		HLA-A*25:01	/	/
		HLA-B*18:01	psoriatic arthritis, psoriasis, type 1 diabetes	NA
		HLA-B*41:01	/	/
		HLA-C*12:03	/	/
		HLA-C*17:01	/	/
		HLA-DPA1*01:03/DPB1*02:01	/	/
		HLA-DPA1*01:03/DPB1*09:01	systemic sclerosis	Japanese
		HLA-DPA1*02:01/DPB1*02:01	/	/
		HLA-DPA1*02:01/DPB1*09:01	/	/
		HLA-DQA1*01:02/DQB1*02:02	multiple sclerosis	Caucasian
			psoriasis	Croatian
			systemic lupus erythematosus	Mexican American
			type 1 diabetes	NA
			Crohn’s disease	Japanese
			primary biliary cirrhosis	Japanese
			celiac disease	Japanese
		HLA-DQA1*01:02/DQB1*06:02	as above except for celiac disease	
		HLA-DQA1*03:03/DQB1*02:02	Graves’ disease	Japanese
			celiac disease	Japanese
			type 1 diabetes	NA
		HLA-DQA1*03:03/DQB1*06:02	Graves’ disease	Japanese
			multiple sclerosis	Cantonese, Caucasian, and African Brazilian
			systemic lupus erythematosus	Mexican American
			type 1 diabetes	NA
			sarcoidosis	Caucasian
		HLA-DRB1*04:05	optic neuritis	NA
			autoimmune disease	Tunisian
		HLA-DRB1*15:01	multiple sclerosis	African American, Caucasian, Australian, and Israeli
			anti-gbm disease	Chinese and Japanese
			uveitis	Japanese
			rheumatoid arthritis	NA
			type 1 diabetes	NA
			systemic lupus erythematosus	Mexican American
			sarcoidosis	NA
		HLA-DRB4*01:03	/	/
		HLA-DRB5*01:01	autoimmune disease	NA
			multiple sclerosis	Caucasian
KPQSAVYSTGSNGILL	CHL1	HLA-A*24:02	arthritis	Basque
		HLA-A*30:01	/	/
		HLA-B*13:02	/	/
		HLA-B*35:08	/	/
		HLA-C*04:01	pemphigus vulgaris	Iranian
		HLA-C*06:02	psoriasis	Korean, Croatian, Caucasian, Chinese, Jew, Spanish, and Swedish
			ankylosing spondylitis	NA
			polyarthritis	NA
			psoriatic arthritis	NA
			pemphigus vulgaris	Iranian
			vitiligo	Chinese
		HLA-DPA1*01:03/DPB1*03:01	iridocyclitis	NA
			ankylosing spondylitis	NA
			arthritis	Caucasian
			pulmonary fibrosis	Korean
			type 1 diabetes	NA
		HLA-DPA1*01:03/DPB1*04:02	type 1 diabetes	NA
		HLA-DQA1*02:01/DQB1*02:02	type 1 diabetes	NA
		HLA-DRB1*07:01	/	/
		HLA-DRB4*01:03	/	/
PPEAEVQALILLDEE	ZHX2	HLA-DRB1*11:01	Lyme arthritis	NA
			colitis	NA
			psoriasis	Iraqi
		HLA-DRB1*15:01	multiple sclerosis	African American, Caucasian, Australian, and Israeli
			anti-gbm disease	Chinese and Japanese
			uveitis	Japanese
			rheumatoid arthritis	NA
			type 1 diabetes	NA
			systemic lupus erythematosus	Mexican American
			sarcoidosis	NA
		HLA-DQB1*03:01	colitis	NA
			reactive arthritis	NA
			pemphigus vulgaris	Non-Jewish
			systemic sclerosis	NA
			vitiligo	Italian
		HLA-DQB1*06:02	multiple sclerosis	Cantonese, African, Brazilian, and Caucasian
			type 1 diabetes	NA
			systemic lupus erythematosus	Mexican American
			sarcoidosis	Caucasian
		HLA-DPB1*01:01	celiac disease	NA, Italian
			Graves’ disease	NA
		HLA-DPB1*04:01	Takayasu arteritis, type 1 diabetes	Japanese
			anti-gbm disease	Chinese
			type 1 diabetes	Saudi Arabian

**Table 4:** Binding affinity of MHC-restricted epitopes of BNT-162b2 vaccine that cross-react with self-antigens according to TepiTool analysis. A lower percentile rank indicates a higher binding affinity. Abbreviations: CHL1, neural cell adhesion molecule L1-like protein; HLA, human leukocyte antigen; MEAF6, MYST/Esa1-associated factor 6; ZFHX2, zinc finger homeobox protein 2.

Cross-Reactive Antigen	Peptide Start	Peptide End	Peptide	Consensus Percentile Rank	Allele/Haplotype
MEAF6	6	14	MYGNIIRGW	0.1	HLA-A*24:02
	9	17	NIIRGWDRY	0.46	HLA-A*25:01
	10	18	IIRGWDRYL	0.94	HLA-C*12:03
	8	22	GNIIRGWDRYLTNQK	0.07	HLA-DPA1*01:03/DPB1*02:01
	1	15	LEDTQMYGNIIRGWD	0.004	HLA-DPA1*01:03/DPB1*02:01
	1	15	LEDTQMYGNIIRGWD	0.31	HLA-DQA1*01:02/DQB1*06:02
	8	22	GNIIRGWDRYLTNQK	0.03	HLA-DQA1*01:02/DQB1*06:02
	8	22	GNIIRGWDRYLTNQK	0.01	HLA-DRB1*04:05
	1	15	LEDTQMYGNIIRGWD	0.005	HLA-DRB1*04:05
	8	22	GNIIRGWDRYLTNQK	0.28	HLA-DRB1*15:01
	1	15	LEDTQMYGNIIRGWD	0.13	HLA-DRB1*15:01
CHL1	6	14	VYSTGSNGI	0.31	HLA-A*24:02
	2	16	PQSAVYSTGSNGILL	0.75	HLA-DRB1*07:01
ZHX2	1	15	PPEAEVQALILLDEE	0.002	HLA-DRB1*11:01
	1	15	PPEAEVQALILLDEE	0.004	HLA-DRB1*15:01

**Table 5:** Summary of key studies reporting the association between HLA alleles or haplotypes and COVID-19 severity or vaccine response in different geographic areas. Abbreviations: COVID-19, CoronaVirus Disease-19; HLA, human leukocyte antigen; MIS-C, multisystem inflammatory syndrome in children; RA, rheumatoid arthritis; SARS-CoV-2, severe acute respiratory syndrome coronavirus 2; UK, United Kingdom; US, United States.

Author, Year	Ethnic Group	Number of Patients or Samples	HLA Allele	Outcome
Astbury et al., 2022 [39]	UK cohort of European and non-European descendance	1364	HLA-DRB1*13:02	Symptomatic COVID-19
			HLA-DRB1*15:02	Less T-cell responsiveness to both SARS-CoV-2 spike and nucleoprotein
			HLA-DRB1*04:04 HLA-DRB1*07:01	Lower anti-spike antibody titer in previously infected individuals receiving one single dose of BNT-162b2 vaccine
			HLA-DRB1*03:01	Higher anti-spike antibody titer in previously infected individuals receiving one single dose of BNT-162b2 vaccine
			HLA-DRB1*15:01	4–6-fold enhancement of anti-spike T-cell response in previously infected individuals receiving one or two doses of BNT-162b2 vaccine
Sacco et al., 2022 [40]	US	262	HLA-A*02	Higher risk of MIS-C in SARS-CoV-2-infected children
			HLA-B*35	
			HLA-C*04	
Bertinetto et al., 2023 [41]	Italian	416	HLA-A*03:01 HLA-B*40:02 HLA-DPB1*06:01	Higher anti-spike humoral response in BNT-162b2 vaccine-recipients
			HLA-A*24:02 HLA-B*08:01 HLA-C*07:01	Lower anti-spike humoral response in BNT-162b2 vaccine-recipients
			HLA-DRB1*15:01 HLA-DRB1*13:02	Higher anti-spike T-cell response in BNT-162b2 vaccine-recipients
			HLA-DRB1*11:04	Lower anti-spike T-cell response in BNT-162b2 vaccine-recipients
Higuchi et al., 2023 [42]	Japanese	87	HLA-DRB1*12:01 HLA-DQB1*03:01	Higher humoral response in RA patients vaccinated with BNT-162b2 mRNA formulation
			HLA-DRB1*15:01 HLA-DQB1*06:02	Higher production of neutralizing antibodies in RA patients vaccinated with BNT-162b2 mRNA formulation
Romero-Lopez et al., 2021 [43]	Mexican	5840	HLA-DRB1*01	Lower rate of COVID-19 fatal cases in carriers of this HLA allele
Pisanti et al. 2020 [44]	Italian	104,135	HLA-A*01:01g-B*08:01g-C*07:01g-DRB1*03:01g	Higher COVID-19 morbidity and mortality rates among inhabitants of northern regions of Italy
			HLA-A*02:01g-B*18:01g-C*07:01g-DRB1*11:04g	Lower COVID-19 morbidity and mortality rates among inhabitants of southern regions of Italy

**Table 6:** Summary of the main studies investigating the safety of COVID-19 vaccines in patients with autoimmune rheumatic diseases. Abbreviations: AEs, adverse events; HCs, healthy controls; PsO, psoriasis; SARS-CoV-2, severe acute respiratory syndrome coronavirus 2; SLE, systemic lupus erythematosus; SLEDAI-2K, systemic lupus erythematosus disease activity index 2000; UK, United Kingdom.

Author, Country, Year	Type of Study	Population	Disease	Type of Vaccine Administered	Results
Simoncelli et al., Italy, 2023 [5]	multicenter case–control study	107 patients and 107 HCs	systemic vasculitis	mRNA or viral vector vaccine	Only one flare of microscopic polyangiitis after the 1st dose of mRNA vaccine reported; no significant differences between patients and HCs in the rate of AEs in the other cases
Vacchi et al., Italy, 2022 [6]	multicenter retrospective study	416 patients (92.3% of whom were vaccinated)	mixed cryoglobulinaemic vasculitis	mRNA or viral vector vaccine	Mild and self-limiting AEs recorded in 31.7% of cases; vasculitis flares recorded in 5.3% of patients mainly suffering from peripheral neuropathy or skin vasculitis
Mohanasundaram et al., India, 2022 [7]	multicenter, cross-sectional study	2092 patients (61.8% of whom were vaccinated)	miscellaneous	viral vector and inactivated SARS-CoV-2 vaccine	AEs reported in 33.64% of vaccine recipients, being mainly fever and myalgia; disease flares following vaccination reported in 2.47% of recipients
Yuki et al., Brazil, 2022 [11]	prospective controlled study	232 SARS-CoV-2-naive SLE patients and 58 SARS-CoV-2-naive HCs	SLE	inactivated SARS-CoV-2 vaccine	Arthralgia after the first dose more common in SLE pts vs. controls (14.7% vs. 3.4%); myalgia after the second dose less common in SLE pts vs. controls (6.5% vs. 15.5%); SLE flares reported in 4.7% of the patients after full immunization; no worsening of SLEDAI-2K score up to 3 months after full vaccination
Zavala-Flores et al., Peru, 2022 [8]	descriptive observational study	100 patients	SLE	mRNA vaccine	AEs reported in 92.2% of vaccinated patients, being mostly local and of mild intensity; 27 post-vaccination flares (arthritis and skin rashes); the use of hydroxychloroquine and a history of renal disease were associated with a lower risk of presenting post-vaccination flare
Tzioufas et al., Greece, 2021 [12]	prospective multicenter study	605 patients and 116 HCs	systemic autoimmune and autoinflammatory rheumatic diseases	mRNA vaccine	Clinical deterioration observed by clinicians in 10.57% patients; self-reported disease worsening in 15% of patients; similar rate of mild AEs between patients and HCs
Shinjo et al., Brazil, 2022 [9]	prospective controlled study	53 patients and 106 HCs	idiopathic inflammatory myopathies	inactivated SARS-CoV-2 vaccine	Similar frequencies of AEs between patients and HCs
Furer et al., Israel, 2022 [10]	multicenter observational study	710 patients and 124 HCs	miscellaneous	mRNA vaccine	Similar prevalence rate of mild AEs in patients and controls; stable disease activity scores following vaccination; 2 cases of death, 6 cases of non-disseminated herpes zoster infection, 2 cases of uveitis, and 1 case of pericarditis occurring in vaccinated patients
Nakafero et al., 2023, UK [45]	self-controlled case series	3554 patients	miscellaneous	mRNA or viral vector vaccine	No disease flares recorded after vaccination
Mahil et al., 2022, UK [46]	longitudinal cohort study	67 patients and 15 HCs	PsO	BNT-162b2 mRNA vaccine	PsO flare in 12% of patients; T-cell responses recorded in a lower percentage of immunosuppressed patients compared with controls

## Data Availability

The data presented in this study are available in Supplementary Materials.

## Supplementary Materials

The following supporting information can be downloaded at: https://www.mdpi.com/article/10.3390/microorganisms11071686/s1, Table S1: List of IEDB epitopes corresponding to the FASTA protein sequence of BNT-162b2 vaccine as of 7 May 2023.
